# Supplementary material for: Analysis of Prognostic Factors for Internal Carotid Artery Invasion by Nasopharyngeal Carcinoma
Source: Cancers (Basel). 2025 Feb 1;17(3):488. doi: 10.3390/cancers17030488 (PMC11816255; doi:10.3390/cancers17030488)
Supplement: Supplementary file 1 [file cancers-17-00488-s001.zip › cancers-3445277-supplementary.pdf]

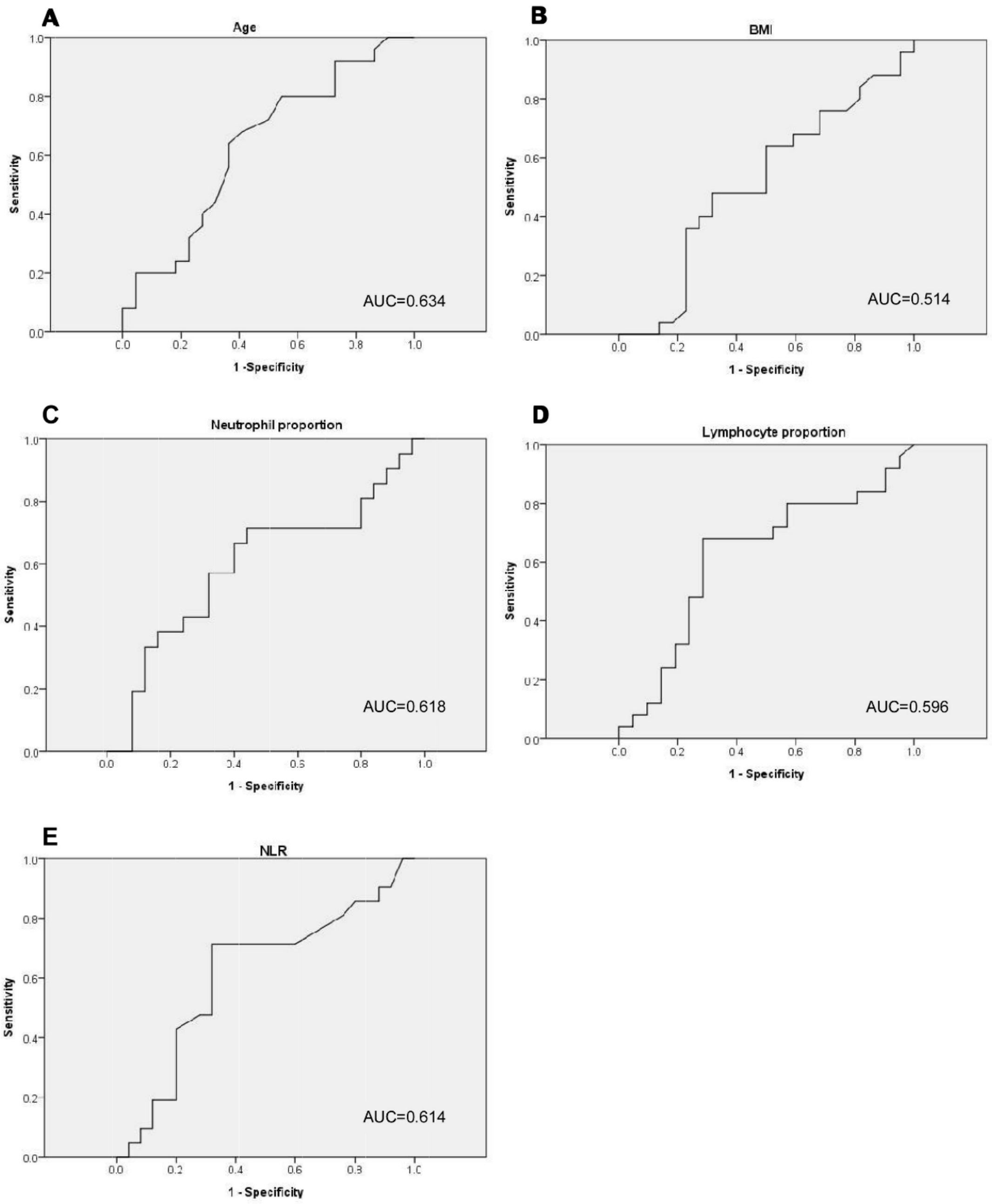

Supplementary Figure S1. Receiver operation characteristic analysis curves for poor vs. good treatment response in NPC patients with ICA invasion using (A) age, (B) body mass index (BMI), (C) neutrophil proportion, (D) lymphocyte proportion, and (E) neutrophil-to-lymphocyte ratio (NLR).
